# Underwater Structured Light Stripe Center Extraction with Normalized Grayscale Gravity Method

**DOI:** 10.3390/s23249839

**Published:** 2023-12-15

**Authors:** Shuaishuai Li, Xiang Gao, Zexiao Xie

**Affiliations:** 1 College of Engineering, Ocean University of China, Qingdao 266100, China; lss7060@stu.ouc.edu.cn; 2 Key Laboratory of Ocean Engineering of Shandong Province, Qingdao 266100, China; 3 Institute of Automation, Chinese Academy of Sciences, Beijing 100190, China

**Keywords:** light stripe center extraction, underwater measurement, underwater semantic segmentation, normalized grayscale gravity method

## Abstract

The non-uniform reflectance characteristics of object surfaces and underwater environment disturbances during underwater laser measurements can have a great impact on laser stripe center extraction. Therefore, we propose a normalized grayscale gravity method to address this problem. First, we build an underwater structured light dataset for different illuminations, turbidity levels, and reflective surfaces of the underwater object and compare several state-of-the-art semantic segmentation models, including Deeplabv3, Deeplabv3plus, MobilenetV3, Pspnet, and FCNnet. Based on our comparison, we recommend PSPnet for the specific task of underwater structured light stripe segmentation. Second, in order to accurately extract the centerline of the extracted light stripe, the gray level values are normalized to eliminate the influence of noise and light stripe edge information on the centroids, and the weights of the cross-sectional extremes are increased to increase the function convergence for better robustness. Finally, the subpixel-structured light center points of the image are obtained by bilinear interpolation to improve the image resolution and extraction accuracy. The experimental results show that the proposed method can effectively eliminate noise interference while exhibiting good robustness and self-adaptability.

## 1. Introduction

In recent years, deep learning and intelligent devices have become increasingly ubiquitous, especially in the fields of marine bioengineering, marine environmental protection, and underwater target detection. Research on underwater detection is gaining increasing importance due to the complex underwater environment, diverse underwater species, and related issues. Active vision techniques are particularly crucial for underwater detection, and there is a growing need to develop and utilize these techniques to enhance detection capabilities. Therefore, high-quality research in this area is in demand to advance state-of-the-art underwater detection technology. Line structured light technology is a typical active vision measurement method [1], which is widely used for non-contact precision measurement of geometric parameters due to its flexibility and accuracy.

Depending on the measurement distance and equipment, line-structured laser stripes are usually several to tens of pixels wide when acquired, and center extraction is an important step in the measurement process [2]. According to Steger [3], for a lens with a focal length of 12 mm and a distance of 50 cm from the workpiece, each deviation of 0.1 pixels in the image results in a shift of 50 μm in practice. Therefore, the center extraction of a laser stripe at the pixel level is a challenge to meet the requirements for high measurement accuracy. Many researchers have investigated center extraction methods with the goal of achieving better accuracy, higher efficiency, greater robustness, and lower noise. Depending on the minimum coordinate value, these algorithms can be divided into two categories: pixel-level center extraction and sub-pixel-level center extraction. The pixel level includes the extreme-value method, the threshold method, and the directional-template method. The other category includes the grayscale gravity method, curve fitting, and the Hessian-matrix method.

The method of extreme value [4] selects the maximum pixel on the section as the center point, which has low accuracy and is sensitive to noise. When extracting the cross-section of laser stripes using the threshold methods [5], identification errors can occur due to asymmetric grayscale distributions or the influence of noise. The improved adaptive grayscale threshold method [6] and variable threshold centroid method [7] can alleviate the limitations of conventional approaches, but the extraction accuracy still falls short of expectations. The direction template and the improved direction template methods [8,9] can also obtain the stripe center at the pixel level, and the influence of noise on the center line extraction can be suppressed effectively, but these methods do not yield satisfactory results when applied to irregular laser stripes and require a large amount of calculation due to the cross-correlation processes.

The grayscale gravity method [10] is based on the characteristics of the grayscale distribution within the cross-section of each row of laser stripes and extracts the grayscale centroid of the laser stripe region by computing line-by-line in the direction of the line coordinates. This reduces the error caused by the uneven grayscale of the laser stripes and is fast, but requires that the grayscale is Gaussian-distributed and susceptible to noise. The curve fitting method [11] uses a Gaussian curve or parabola to sketch the grayscale distribution of the laser stripe’s cross-section. The center of the cross-section is the local maximum of the fitted curve; this method is only applicable for wide laser stripes with constant normal vector direction. In addition, the actual grayscale distribution of the pixels in the laser line is not strictly symmetrical, so the extreme points found by curve fitting are often not in the actual center of the laser line. The Hessian-matrix method [12,13] of the laser stripe normal vector direction is derived from the eigenvectors corresponding to the eigenvalues of the Hessian-matrix. The subpixel center coordinates of the laser line are calculated by applying the Taylor expansion in the normal vector direction. The Hessian-matrix method is characterized by high noise insensitivity, accurate extraction, and good robustness, and it has obvious advantages under the conditions of complex environments and high precision requirements. However, it generates redundant center points in wider cross-sections and is less satisfactory because of the high real-time computational cost.

In complex underwater measurement environments, it is difficult to ensure that the energy distribution and morphological characteristics of the stripes remain stable, and these stripes are often influenced by the reflectance properties of objects, resulting in variable energy and morphology at different locations. Therefore, in this paper, based on the traditional methods, we propose a grayscale normalization method. First, the structured light stripe is extracted using the semantic segmentation method to determine the region of interest, which can effectively avoid the interference of Fresnel scattering on the outside of the light stripe and the influence of noise that cannot be avoided by the thresholding method. Finally, the center of the subpixel light stripe of the region of interest is determined by normalizing the grayscale values.

## 2. Background

In complex underwater environments, when the laser illuminates an object’s surface, speckles (Fresnel scattering [14]) are generated in the nearby free space with regard to the light field at any point on the screen, and the wavelets, which are generated by all scattering sources from the entire diffusing surfaces, are superimposed, which then has a noisy effect on the original laser stripe. Depending on the reflective properties, the surface of the object can be divided into a Lambert surface, a mirror surface, and a mixed reflective surface (weakly scattering surface), whose reflective properties are in between the above two. If there are multiple components of reflected light from the scattering surface, the observed surface scattering is a superposition of these partial light fields, according to the Phong [15] hybrid model:

(1) $$I=k_{\alpha }I_{\alpha }+k_{d}I_{pd}\cos\theta +k_{s}I_{\mathrm{ps}}{\left(\cos\phi \right)}^{n},$$

where *I* is the brightness of the received reflected light, $I_{\alpha }$ is the reflected ambient component, $I_{pd}$ is the diffuse component, $I_{ps}$ is the specularly reflected component, $k_{\alpha }$, $k_{d}$, and $k_{s}$ are the reflection coefficients of ambient, diffuse, and specular light, respectively, ϕ is the viewing angle and direction of the specularly reflected light, *n* is the specular refractive index, and $I_{p}$ is the intensity of the illumination source.

**Figure 1:**
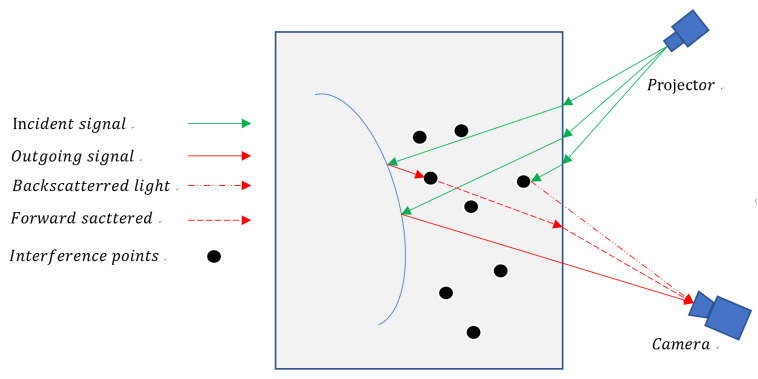
Underwater light transmission.

The images taken underwater have a significant loss in contrast and brightness due to underwater floating objects, etc. The underwater light transmission model is shown in Figure 1. The light that the detector receives includes direct light, back-scattering light, and forward-scattering light. The captured intensity of the camera becomes:

(2) $$J_{n}^{D}\left(x,y\right)=T_{1}\left(x,y\right)\times I_{n}^{C}\left(x,y\right),$$

where $T_{1}\left(x,y\right)$ refers to the medium transmittance of turbid water. $I_{n}^{C}\left(x,y\right)$ denotes the observed value of the color image or the observed value of a particular channel of the image (e.g., red, green, or blue channel) at pixel (x,y) [16].

The forward scattering of light describes the direct light scattered by particles before reaching the detector; the forward scattering will blur the direct light. The captured intensity of the camera becomes:

(3) $$\begin{array}{cc} J_{n}^{F}\left(x,y\right) & =\mathrm{conv}\left(I_{n}^{C_{D}},P\right)\\ \; & =\sum_{g=0}^{X-1}\sum_{t=0}^{Y-1}\left[I_{n}^{C_{D}}\left(g,t\right)P\left(x-g,y-t\right)\right], \end{array}$$

where (x,y) is the camera resolution, and P(x,y) denotes the underwater point spread function (PSF).

Since PSF is influenced by water-suspended particles, the PSF models are usually parameterized by choosing various empirical constants as:

(4) $$P\left(x,y\right)=T\left(x,y\right)\times F^{-1}\left[e^{-Q\times z(x,y)\times \omega (x,y)}\right],$$

where $T=T_{2}-T_{1}$, $T_{2}$ is an empirical constant [17] (i.e., $T_{2}>T_{1}$), *Q* is an empirical damping factor related to the water turbidity, $F^{-1}$ denotes the inverse Fourier transform, and ω is the spatial frequency of the captured image in the image plane [18,19].

The backscattering of light describes the direct light scattered by particles before achieving the object’s surface. The backscattering adds specific strongly correlated noise to the direct light and the light of forward scattering. The captured intensity of the camera should be:

(5) $$J_{n}^{B}\left(x,y\right)=\left[J_{n}^{D}\left(x,y\right)+J_{n}^{F}\left(x,y\right)\right]\times G_{n}\left(x,y\right)$$

where $G_{n}$ is the white noise with zero mean and variance.

**Figure 2:**
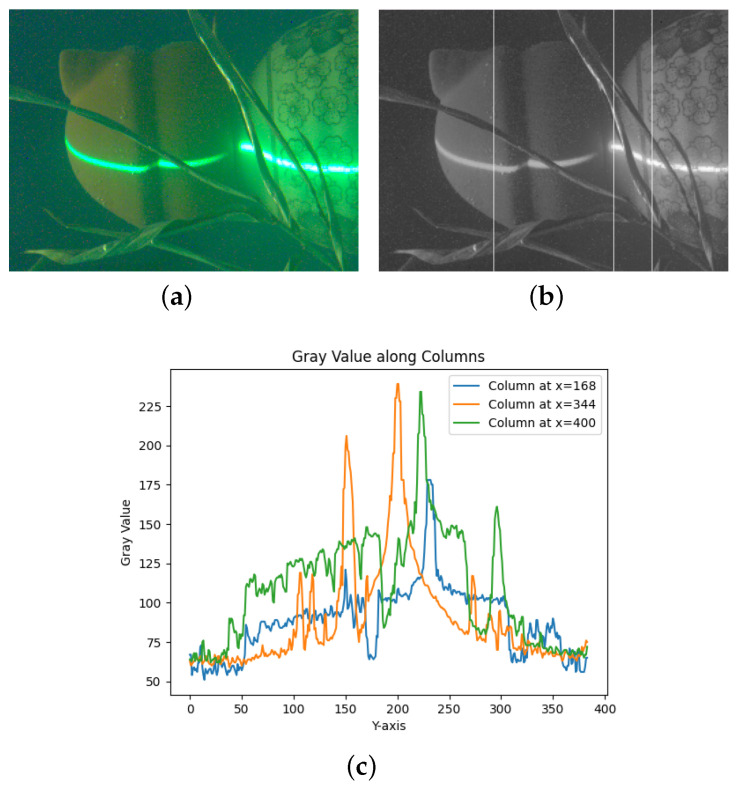
Stripes with cross-sectional grayscale statistics. (**a**) Laser light underwater imaging, (**b**) grayscale light stripe image, (**c**) column orientation grayscale change diagram.

When analyzing the underwater image, the gray levels of different columns along the cross-sectional direction of the laser stripe are extracted and compared, and the gray levels of the image vary significantly when Fresnel scattering is present, as shown in Figure 2.

By extracting the grayscale values of different columns along the cross-sectional direction of the laser stripes—specifically on rough surfaces, transitions, and smooth surfaces—the respective columns selected are the 168th, 344th, and 400th. We analyzed the imaging characteristics of different reflective surfaces, and the grayscale plots for these columns are shown in Figure 2c. From the diagram, it can be seen that surfaces with high absorption have small gray level variations (168th column), which resemble a Gaussian distribution. Fresnel diffraction occurs at the transitions between different objects (344th column), leading to strong scattering at the edges of the stripes and causing significant distortion of the grayscale distribution. On surfaces with high reflectance, the grayscale distribution is strongly affected by noise, resulting in larger fluctuations of the grayscale values. In the case of surfaces that are specularly reflective, strong reflections usually occur when the laser illuminates such an area. The resulting image shows central spots and extended spots in the surrounding regions, as shown in Figure 2a. The size and contrast of the spots are determined by several factors, including illumination, illumination distribution, scattering angle, and distances between surfaces of the medium [20,21].

**Figure 3:**
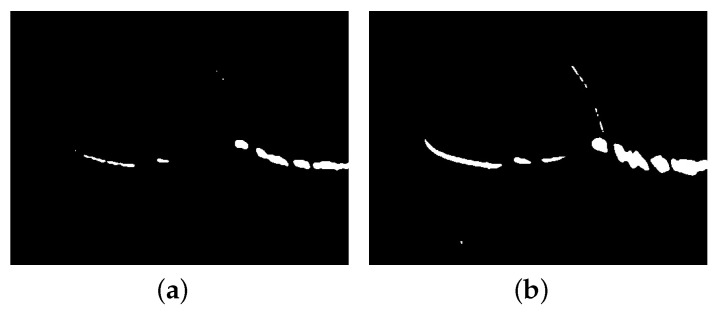
Threshold segmentation after de-noise. (**a**) Segmentation at threshold 170, (**b**) segmentation at threshold 155.

Conventional algorithms such as noise reduction by filtering, threshold segmentation, the difference method, or adding structured light-specific filters to the waterproof cover of the viewing device to eliminate ambient light cannot avoid the influence of speckles. As shown in Figure 3, according to the grayscale distribution in Figure 3, the image is segmented by the grayscale threshold after median filtering. When the threshold is set to 170 (Figure 3a), most of the scattered spots can be eliminated, but for the objects with low reflectivity, most of the laser lines on their surfaces are also masked out. When the threshold is set to 155 (Figure 3b), the laser lines on the surfaces of the objects with low reflectivity are segmented more completely. However, due to the specular reflection, the segmentation of error on the objects with smooth surfaces is large, and the interference caused by the scattered light cannot be eliminated.

**Figure 4:**
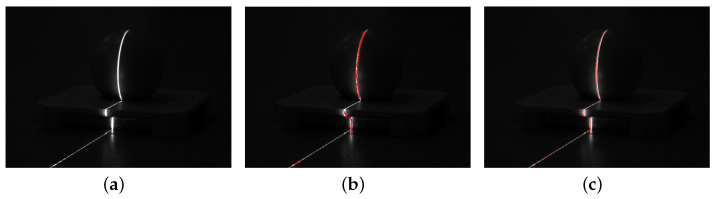
Center extraction after threshold 150 segmentation. (**a**) Original image, (**b**) directional template method, (**c**) grayscale method of gravity.

Among the most popular light bar center extraction methods, both the directional template method and the grayscale center of gravity method for images with scattering need to go through pre-processing of the image, and both need to go through threshold segmentation, although the processing is different. Figure 4 shows the two center extraction effects in the case of threshold 150 segmentation.

When the gray value is greater than 150, its valid light bar information is ignored. As a result, speckles at the edges of light stripes directly affect the position when the center of the stripe is extracted after processing with the conventional threshold algorithm. This leads to detection errors and a rapid increase in measurement inaccuracy.

## 3. Segmentation of Regions of Interest

The regions of interest, as the name implies, are the actual areas that need to be operated. In this paper, they represent the structured light stripes on the surface of the object to be measured by effectively extracting the regions of interest; unnecessary computations of the grayscale values in other areas can be reduced, improving the computational speed of extracting the light stripe centers. When using the normalized grayscale gravity method to calculate the light stripe center, the influence of noise in the background region is avoided; in addition, the speckle points around the specular reflections are effectively eliminated, which increases the accuracy of the center extraction.

**Figure 5:**
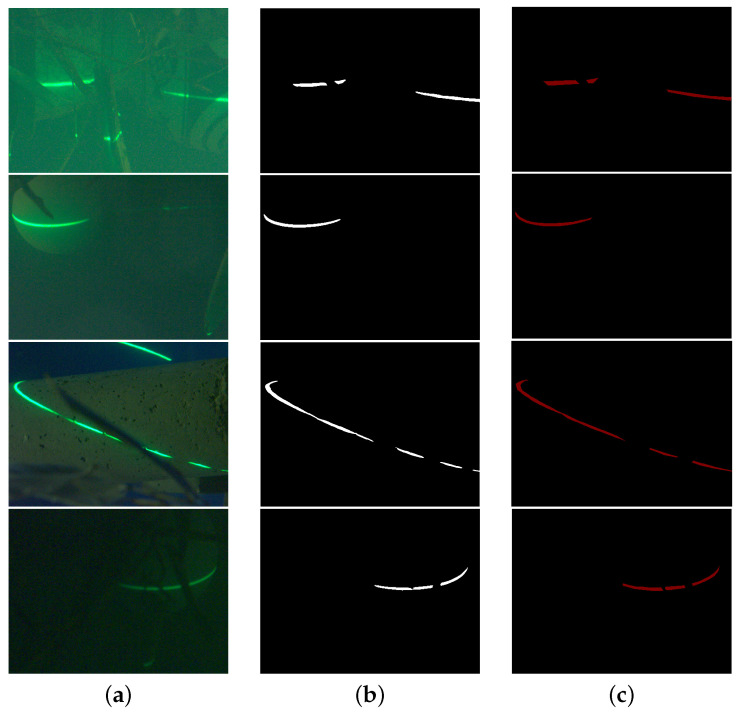
Four semantic segmentation instances and corresponding ground truth. (**a**) Original image, (**b**) predicted labels, (**c**) ground truth.

Semantic segmentation is a well-studied problem in robot vision and deep learning [22,23] because it is practical and effective in estimating scene geometry, inferring interactions and spatial relationships between objects, and detecting target objects, which plays an important role in extracting structured light regions of interest in underwater machine vision. Currently, manually labeled datasets, such as ImageNet [24], ADE20K [25], PASCAL [26], and COCO [27], are playing a significant role in improving image processing tasks and driving research in new directions. Datasets with underwater images, such as SUIM [28] or Seagrass [29], are intended for semantic segmentation tasks of the classification of fish or marine life. In this paper, a large number of underwater images of green light stripes are acquired, which include the test object, the green light stripes projected on the test object, the background, and the green light stripes projected on the background; however, only the light stripe on the object under test is the actual area of interest in the entire image. As shown in Figure 5, we create the underwater structured light dataset (USLD) from multiple viewpoints for different underwater turbidities, different lighting environments, and different targets and label the foreground structured light as the region of interest, which contains a total of 860 RGB images. The percentages of specific classifications are shown in Table 1. For the sake of description, the test object and the light stripe on the test object are referred to as the foreground and foreground light stripes, respectively. The area outside the test object is the background, and the background is divided into pure background and background light stripes. The laser light stripes on the target are labeled as GS (Green_stripe), and the rest are labeled as BG (Background). The purpose of semantic segmentation is to extract the laser stripes on the target effectively and determine the region of interest for subsequent extraction of the laser stripe’s centroid.

In this article, several state-of-the-art models for deep convolutional networks are presented. CNN models are generally divided into encoders and decoders. Encoders typically use backbone networks to extract features and generate feature maps that contain semantic information about the input image that can be used in subsequent decoding and segmentation tasks. During the evaluation in this work, some segmentation models were used multiple times but with different backbone networks. The complete list of models can be found in Table 2.

During the training process, more training data were generated by rotating, flipping, scaling, and horizontally flipping the dataset, which enables the network to better evaluate the learning and enhance its generalization ability. To assess the segmentation effects of different network structures, all models were implemented in Python using the PyTorch library, and a server equipped with two NVIDIA 3090 GPUS was used to ensure consistent hardware configuration across all models.

To evaluate the correctness of the pixel-by-pixel classification, two supervised evaluation methods were utilized: the intersection over union (IoU) and the F1 score. The former, also known as the Jaccard Index, is one of the most used metrics for semantic segmentation tasks; it consists of the area of overlap between the predicted masks and the ground truth divided by the area of union between the prediction and the ground truth

(6) $$\begin{array}{cc} \mathrm{IoU} & =\frac{\;\mathrm{Area}\;\mathrm{of}\;\mathrm{overlap}\;}{\;\mathrm{Area}\;\mathrm{of}\;\mathrm{union}\;}\\ \; & =\frac{\;\mathrm{TP}\;}{\;\mathrm{TP}\;+\;\mathrm{FP}\;+\;\mathrm{FN}\;} \end{array}$$

In the context of evaluation, TP refers to the true positive cases where the model correctly predicts a positive case, TN refers to the true negative cases where the model correctly predicts a negative case, FP refers to the false positive cases where the model falsely predicts a negative case as a positive case, and FN refers to the false negative cases where the model falsely predicts a positive case as a negative case. It is also regarded as a region similarity metric.

The latter is also called the dice coefficient and provides the contour accuracy F1

(7) $$F1=\frac{2\times P\times R}{P+R}$$

It is defined as the harmonic mean of the precision P and recall R of the model. P is the ratio of the number of correctly classified samples to the total number of samples, and R is the ratio of the number of correctly classified samples to the actual number of positive samples.

In addition, this work considers that there is a certain time limit for the practical application of the extraction of the light stripe center, so the inference time must be taken into account, and for the real-time function, at least 15 frames per second (FPS) are required.

**Figure 6:**
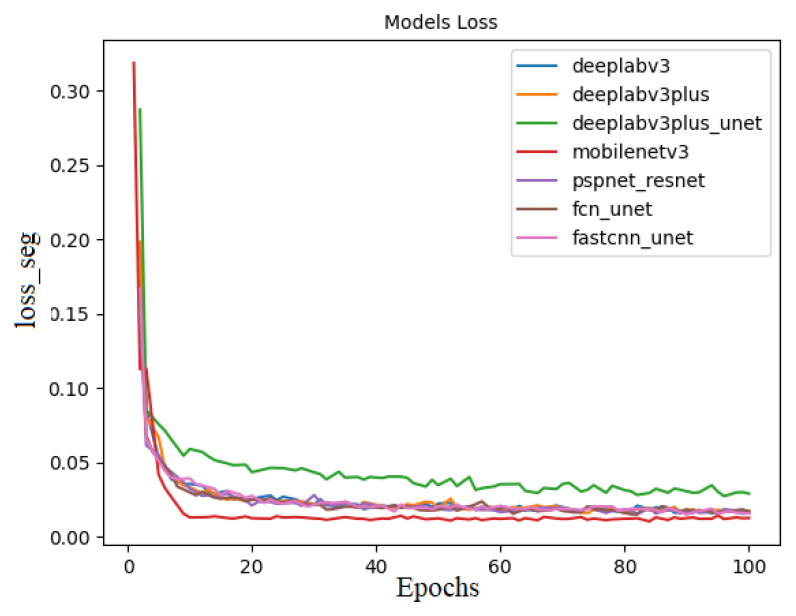
Training loss over epochs of each model.

**Figure 7:**
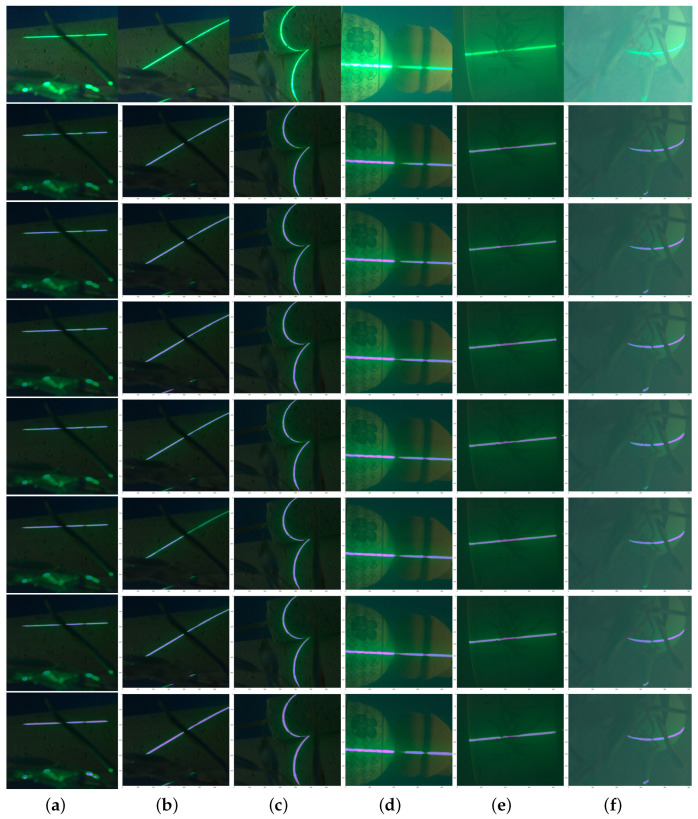
Semantic segmentation instances: from top to bottom, they represent the original graph, Deeplabv3, Deeplabv3plus, Deeplabv3plus+unet, MobilenetV3, Pspnet+resnet, Pspnet+unet, and FCNnet+unet semantic segmentation model.

Baseline evaluations with state-of-the-art deep learning segmentation models show that good results (Table 3) can be obtained for all selected models. Moreover, all models show similar convergence behavior, as shown in Figure 6. Table 3 presents the results of the benchmark evaluation. According to the table, Pspnet using a ResNet101 backbone provides the best results for underwater laser line segmentation with an average IoU of 88.95 and an average F1 score of 93.80. The inference time of the FastSCNN model shows the best time of 43.8 FPS. The visualization of the test set, according to the above model, to segment the image is shown in Figure 7.

In determining the region of interest, we attempted to circumvent certain limitations by providing a dataset for semantic segmentation of underwater laser line images, and benchmark evaluation showed that the Pspnet segmentation model with a Resnet101 backbone gave the best overall performance in terms of segmentation results and inference time, making it a good candidate for the next step of the work. Determining the region of interest through semantic segmentation can effectively exclude background light stripes to reduce computational effort and avoid underwater scattering spot and noise interference that cannot be handled by traditional segmentation.

Accurate and complete segmentation of the region of interest is crucial to prepare for subsequent matching and measurement tasks.

## 4. Normalized Grayscale Gravity Method

### 4.1. Laser Stripe Characteristics

**Figure 8:**
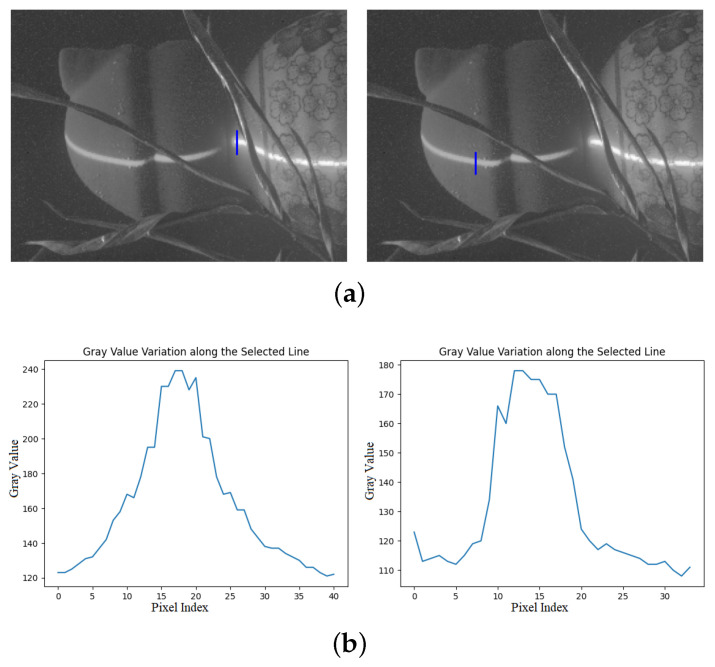
Grayscale distribution of different cross-sections. (**a**) Selection of cross-section, (**b**) grayscale intensity distribution.

Since the plane of light formed by the line laser emitter has a certain thickness, and the intersection between the laser and the surface of the object under examination has a certain width, the light reflected from the object is called a laser stripe. The majority of studies concluded that the laser stripe obeys a Gaussian distribution, and the center of the cross-section of the stripe is the brightest region, gradually decreasing toward the sides. However, in a real environment, the distribution of the laser stripes on the cross-section does not fully conform to the Gaussian distribution due to external environmental influences, the smoothness of the surface of the reflected object, and the properties of the laser itself, as shown in Figure 8.

**Figure 9:**
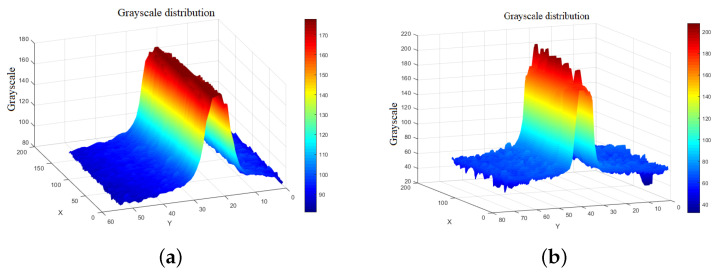
Grayscale of surfaces with different roughnesses. (**a**) Laser stripe on smooth planes, (**b**) laser stripe on rough planes.

The laser stripe extraction center was selected at different positions in the form of pixels or pixel blocks and was performed row-by-row or column-by-column, as shown in Figure 8. The gray cross-section of the stripe does not comply with the Gaussian distribution due to the superposition of the laser’s second-order diffraction scattering, which leads to multiple saturation of the image and the appearance of a gray distribution jump. The laser stripe center region has high energy, and the upper part of the Gaussian curve is flattened. There is a significant difference in the distribution of grayscale between a uniform, smooth surface and a rough surface, which is shown in Figure 9. This three-dimensional illustration demonstrates that in a uniform, smooth plane, the grayscale distribution changes less, and the energy is mainly concentrated in the center. However, on a rough surface, the grayscale changes significantly, and the energy distribution is extremely uneven. This is due to diffuse scattering on the rough surface, which causes an unbalanced capture of light by the camera lens. Comparing the two images, the center part of the light stripe is flatter in the rough plane, the energy distribution is more widely scattered, and the jump in the cross-section is larger, which requires higher accuracy for extracting the center of the light stripe.

### 4.2. Extraction of Sub-Pixel Center

#### 4.2.1. Grayscale Threshold Method

**Figure 10:**
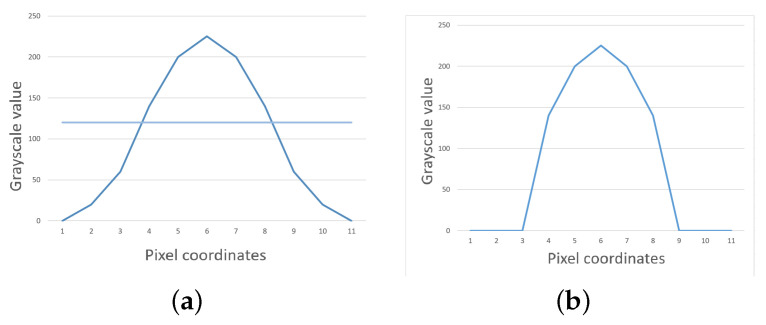
Grayscale threshold segmentation illustration. (**a**) Before threshold segmentation, (**b**) after threshold segmentation.

The grayscale gravity method is based on the grayscale distribution characteristics within the laser stripe cross-section of each row or column of pixels on a pixel-by-pixel basis. The position of the center of the laser stripe of the cross-section is obtained by grayscale weighting the pixel coordinates. Finally, all the centers are adjusted to form the centerline of the laser stripe. The grayscale gravity method is one of the methods for sub-pixel extraction of stripe geometric centers, which has better extraction accuracy for laser stripes with uniform energy distribution and is operated as follows:

1. Image conversion to grayscale and filtering (Gaussian filtering, median filtering, etc.)
2. Determine the region of interest of the image to extract laser grayscale stripes.
3. Select a threshold value in the laser stripe cross-section as the segmentation threshold, as shown in Figure 10.
4. The grayscale gravity method is used to calculate the center of the laser stripe of the threshold segmented; the equation for the coordinates of the center of gravity (*u*, *v*) of the light stripe cross-section is shown in Equation (8).
  (8) $$u=\frac{\sum_{k=0}^{n}\left(h_{k}-T\right)\cdot u_{k}}{\sum_{k=0}^{n}\left(h_{k}-T\right)},\;v=\frac{\sum_{k=0}^{n}\left(h_{k}-T\right)\cdot v_{k}}{\sum_{k=0}^{n}\left(h_{k}-T\right)}$$
  where, in each case, *T* is the dynamic grayscale threshold of the structural light’s stripe image, $u_{k}$,$v_{k}$ are the pixel coordinates of the *k*th pixel in the image, $h_{k}$ is the gray value of the *k*th pixel in the image, *n* is the number of points greater than the threshold value, and the value of *k* is in the range from 0 to *n*.

The grayscale gravity method can reduce the error caused by the uneven grayscale distribution of the optical stripe to a certain extent and improve the accuracy of the extraction of the center of the laser stripe due to the simplicity of the algorithm. The calculation speed is fast and in real-time when the grayscale gravity method is used. However, with different ambient lighting or exposure, the threshold selection is different for each image, and the threshold segmentation method cannot effectively extract the laser stripe features when there is a significant difference in the surface roughness of the test object. The self-adaptability is not good, as shown in Figure 8. The total number of pixel points included in the calculation on each cross-section is different. The clutter points cannot be excluded, resulting in a directional deviation of the extracted coordinates of the center position along the cross-section.

#### 4.2.2. Normalized Grayscale Gravity Method

**Figure 11:**
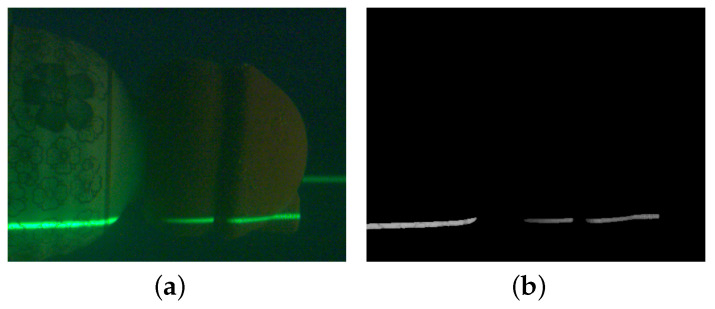
Extraction of grayscale values in the ROI. (**a**) Underwater laser stripe image, (**b**) grayscale image of the ROI.

In this work, we use the semantic segmentation model in determining the region of interest to generate the mask as a binarized image, use the mask to intersect with the original image, retain the grayscale of the original image in the region where the pixel value of the mask image is 1, and set the rest of the region to 0 to create a completely new grayscale image, as shown in Figure 11.

(9) $$C=A\cap B=\left\{x,y|{\left(B\right)}_{x_{1}y_{1}}\cap |\left(A\right)xy\right\}$$

where *A* is the original image, *B* is the semantic segmentation mask corresponding to *A*, ${\left(B\right)}_{x_{1}y_{1}}$ is the pixel coordinate of pixel 1 in the mask, and ${\left(A\right)}_{xy}$ is the pixel coordinate of the original image.

The region of interest in Figure 11b can eliminate the light stripes in the background and extract the grayscale values of object surfaces with different roughnesses. Our proposed method can eliminate the threshold segmentation step while retaining better edge information, which also leads to more extracted information in the cross-section. To reduce the influence of too many edge point grayscale values on the center point, we improve the traditional grayscale gravity method by using the normalized grayscale center of gravity method (10) to strengthen the weighting of extreme values and reduce the weighting of edge points to make the center extraction more robust and converge better.

(10) $$u=\sum_{k=0}^{n}P_{k}\cdot u_{k},\;v=\sum_{k=0}^{n}P_{k}\cdot v_{k}$$

(11) $$P_{k}=\frac{e^{h_{k}-\max\left(h\right)}}{\sum_{k=0}^{n}e^{h_{k}-\max\left(h\right)}}$$

In Equation (11), max(h) is the maximum gray value in the cross-section—the role of which is to prevent exponential overflow—$u_{k}$ and $v_{k}$ are the coordinates of the *k*th pixel in the image, $h_{k}$ is the cross-section of the *k*th pixel gray value, the value of $P_{k}$ is the probability that the grayscale of the current pixel is extreme (0–1), and the more it tends towards 1, the higher the probability of the extreme value. The normalization function allows the conversion of the grayscale values on a given cross-section into a probability distribution in the range [0, 1] with a sum of 1 and the probability that each normalized value after calculation corresponds to an extreme value of the gray level.

**Figure 12:**
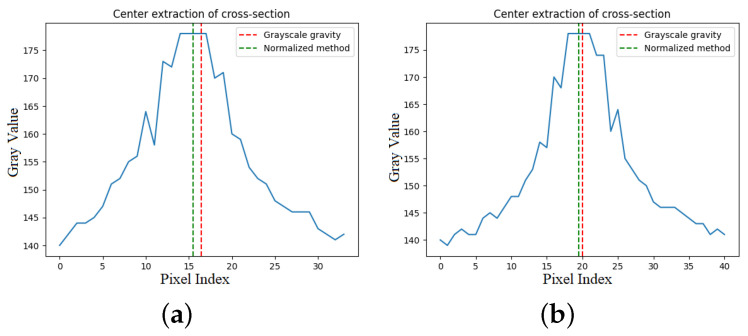
Laser stripe center extraction for different surfaces. (**a**) Rough cross-section, (**b**) smooth cross-section.

The centers of the two methods are found within the cross-section using the traditional grayscale center of gravity method and the improved method, as shown in Figure 12. It is intuitive that the traditional grayscale gravity method is more sensitive to the grayscale values on either side of the peak and that the noise or scattered patches on the surface of the object have a greater impact on the accuracy of the center extraction. In our works, by avoiding threshold segmentation in the light stripe image to retain the original grayscale values, the edge information of the light stripe was also included.

Our proposed normalization of the grayscale using an exponential function normalizes the grayscale values to an exponential growth trend, which means that a small change on the x-axis leads to a large change on the y-axis, and this function curve allows the output values to be kept at a distance, increasing the weights of the grayscale extremes and decreasing or even ignoring the grayscale weights of the boundary points. Then, the grayscale value and pixel coordinates of each row or column are obtained by iteratively going through them and brought into Equations (10) and (11) to calculate the floating-point data value of the laser stripe center coordinates, which is the sub-pixel center point.

The subpixel center points based on each row or column can be found by summing the extreme probabilities of the grayscale since the grayscale values of the subpixel points cannot be determined directly from the two-dimensional image. We apply bilinear interpolation to the image to determine the position of the subpixel to improve the extraction accuracy.

## 5. Experiments and Results

**Figure 13:**
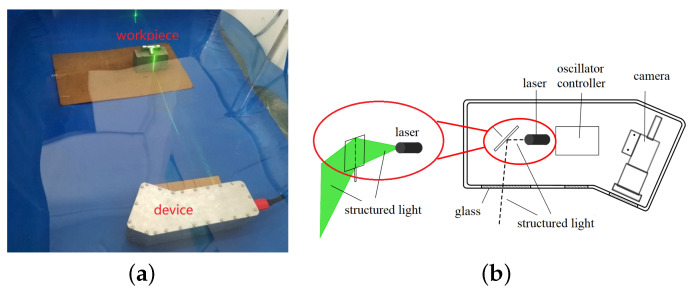
Diagram of laser scanning measuring device. (**a**) Physical picture of device, (**b**) device schematic diagram.

Structured light 3D measurement is a scanning, non-contact survey technique with the advantages of a simple system and high accuracy. An experimental platform for underwater scanning measurements with structured light has been designed, as shown in Figure 13. It mainly consists of a CMOS camera, a lens, a line laser emitter, an oscillator, a D/A converter card, an oscillator controller, and other hardware. The camera selected was an acA1300-200uc industrial camera from Basler, Germany, with a resolution of 1280 (H) × 1024 (V), a pixel size of 4.8μm(H)×4.8μm(V), a chip size of 1/2 CMOS, a frame rate of 200 fps, and selected industrial lens with a focal length of 12 mm. The TS8720 model optical scanning oscillator with a lens thickness of 1 mm was selected, and the input voltage of the oscillator was controlled using a D/A converter card from ADLINK (model 6208) with an input voltage range of −10V∼10V corresponding to an oscillator rotation angle of $-20^{\circ }∼+20^{\circ }$. A green line with a laser wavelength of 532 nm was selected, considering that the water body has the weakest absorption of the blue-green structured light emitter. In this section, the proposed method is implemented in practice in the experimental platform.

With the platform, several comparative experiments were carried out.

**Experiment 1**: Light stripe collection and processing for an uneven-scattering surface.

**Figure 14:**
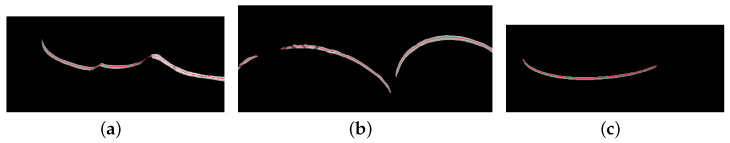
Extraction of sub-pixel centers of laser stripes on the surface of irregular objects. (**a**) object a, (**b**) object b, (**c**) object c.

We used the platform to collect laser stripe images of inhomogeneous surfaces, applied the proposed method to extract the laser stripe centers, and displayed the extracted centers on grayscale images, as shown in Figure 14. The results indicate that our proposed method can accurately detect laser stripe centers on underwater surfaces of different objects and is highly robust under inhomogeneous surface conditions.

**Experiment 2**: Light stripe center extraction and comparison of different methods.

**Figure 15:**
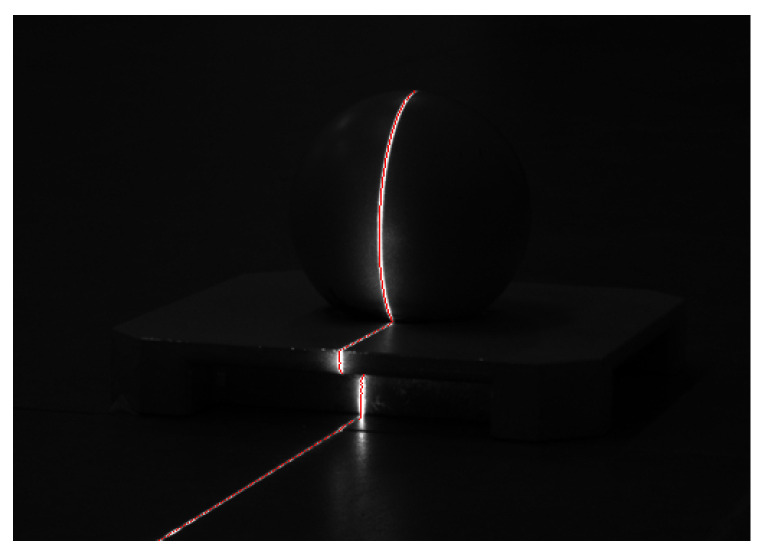
Normalized grayscale gravity method for light stripe center extraction.

Figure 4 shows the inadequacy of conventional methods for laser streak center extraction when flares or scattering spots are produced. In Figure 15, we extract more centers without the need for greyscale thresholding after effectively determining the region of interest by semantic segmentation, and the center extraction via the normalized grayscale gravity method is more convergent and robust compared to the traditional method.

In Figure 15, we extract the light stripes center for a 365 × 520 pixel photo. From Table 4, which shows the comparison of the three different methods, we know that by determining the region of interest by semantic segmentation comparison of thresholding segmentation, the number of center points of the light stripe increases significantly. The template method obviously takes longer and has a larger computational cost, while the remaining two perform well.

**Experiment 3**: Linear comparison of smooth light stripe center extraction.

**Figure 16:**
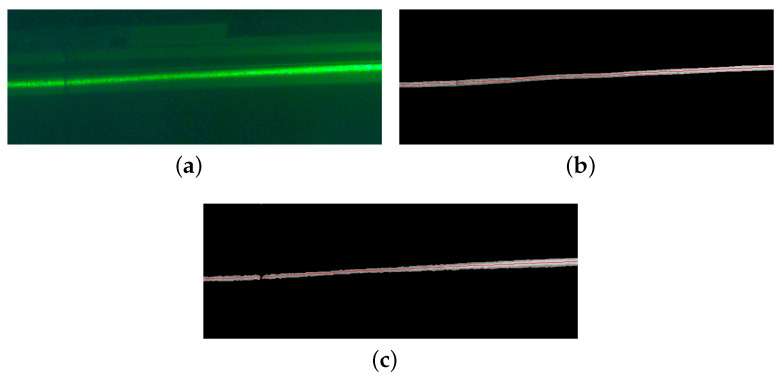
Laser stripe center extraction. (**a**) Light stripe on the standard plane, (**b**) grayscale normalization method, (**c**) grayscale gravity method.

We use the grayscale gravity method and the normalized grayscale gravity method to extract the center of the laser stripes on the surface of planar underwater objects, as shown in Figure 16a. Since the regular surface has good flatness, the coordinates of the line centroids of the regular surface can be used to compare the accuracy of the different algorithms. Figure 16b,c use the normalized grayscale gravity method and the grayscale gravity method, respectively, to extract the center of the laser stripes, both of which can be performed quickly and efficiently.

**Figure 17:**
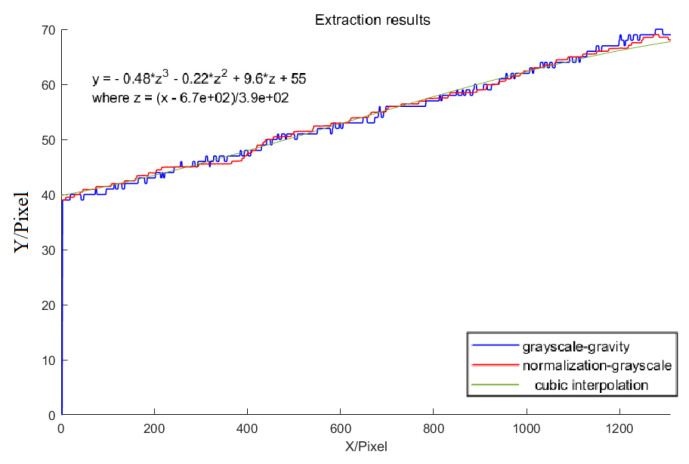
Light stripe on the standard plane.

Figure 17 shows the results of comparing the two methods for optical stripe center extraction. Both the proposed algorithm and the grayscale gravity method operate at the subpixel level. Compared to the traditional grayscale gravity method, the algorithm achieves a more stable center of the laser stripe with less variance, is insensitive to small gray values at the edges, and can converge better in the extreme value region, while the grayscale gravity method achieves more errors in the results due to noise and gray values at the edges.

**Experiment 4**: Repeatability error experiment

The repeatability error (Er) of an algorithm refers to the deviation of the center points extracted at different moments, which reflects the ability of the algorithm to resist random noise at different times. Er can be represented as:

(12) $$Er=\frac{\sum_{j=1}^{p}Er_{j}}{p}$$

where *p* is the total number of laser stripe images taken by the CMOS camera at different moments in time. The error $Er_{j}$ can be obtained by

(13) $$Er_{j}=\frac{\sum_{i=1}^{n}\left|y_{j,i}-y_{1,i}\right|}{n}$$

where y(j,i) is the coordinate of the *i*th center point of the *j*th laser image, and *n* is the number of extracted center points of the laser stripe.

For laser stripe images acquired at different time points, the repeatability error Er was calculated for the two different extraction methods, as shown in Table 5. Compared with the grayscale gravity method, our proposed method retains a smaller repeatability error, so it can extract the centroid of the laser stripe with higher accuracy and reliability.

We also captured images of the extracted stripes at different laser powers to verify the robustness of the method and conducted repeatability error experiments with medium- and low-luminance laser images, using the center of the stripe at the highest luminance image as the reference point.

Table 6 shows that as the laser power changes, the center of the laser stripe also changes to some extent at medium and low power. Compared with the gray center of gravity method, the normalized gray center method has low sensitivity to the laser intensity, which is because the normalized function is extremely sensitive to the change in the gray value, and the extreme value has a large weight on the center point coordinates. The laser stripe edge information or pixel points with low gray values have a negligible weight on the laser center of gravity, which greatly improves the robustness and adaptability of the method.

## 6. Conclusions

Considering the problems of uneven object surface reflection and underwater environment interference during underwater laser measurement, a laser stripe center extraction method based on semantic segmentation with a normalized grayscale gravity method is proposed, which can help the problems of strong underwater environment interference, serious noise, and uneven surface reflection, and so on. The limitations caused by threshold segmentation can be avoided by the semantic segmentation method, which makes it more adaptable. Network training and validation of the state-of-the-art network models are available in Section 3. Pspnet using a ResNet101 backbone can effectively extract the region of interest of the laser stripe. In comparison to the traditional method, the gray normalization method converts gray values into probability distributions of gray extrema. This conversion mitigates the impact of small gray values at the edges and noise, thereby enhancing extraction accuracy. Moreover, the method exhibits superior robustness and generalization ability, as evidenced by the algorithm’s accuracy and stability, as presented in Section 5. It can better complete the extraction and measurement of the center of the laser stripes in the underwater environment.

## Figures and Tables

**Table 1:** Percentage of dataset USLD under different categories.

Classification	Subcategories	Percentage
Turbidity	1.5 NTU	30%
	10 NTU	29%
	15 NTU	24%
	20 NTU	17%
	Rocks	17%
Object Material	Porcelain	73%
	Metalwork	10%
	Low	32%
Camera Exposure	Medium	46%
	High	22%

**Table 2:** Comparison of backbone and parameters of different models.

Model	Backbone	Resolution	FPS
DeeplabV3	Vanilla	512 × 512	17.6
DeeplabV3plus	Xception	512 × 512	29.1
DeeplabV3plus	Unet	512 × 512	14.8
MobilenetV3	MobilenetV3	512 × 1024	33.9
Pspnet	Resnet101	512 × 512	30.4
Pspnet	Unet	512 × 512	31.2
FCN	Unet	256 × 256	13.2

**Table 3:** Comparison of the results of different models.

Model	IOU		*F*1 Score	
	**GS**	**Mean**	**GS**	**Mean**
DeeplabV3	70.09	84.89	82.84	91.13
DeeplabV3plus	71.78	85.74	83.57	91.71
DeeplabV3plus+Unet	74.88	87.29	85.64	92.74
MobilenetV3	62.85	80.19	78.85	86.31
Pspnet+Resnet	78.13	88.95	87.73	93.80
Pspnet+Unet	75.71	87.71	86.18	93.56
FCN+Unet	74.26	86.97	85.23	93.02

**Table 4:** Comparison of three different methods for light stripe center extraction.

	Running Time (ms)	Number of Centers	Stability	Accuracy
Orientation template method	19.2	176	mediocrity	wrong
Grayscale method of gravity	0.45	203	mediocrity	relatively wrong
Normalized grayscale gravity method	0.64	313	relatively good	relatively good

**Table 5:** Repeatability error of extracting at different times.

Method	Grayscale gravity method	Proposed method
Er/pixel	1.2032	0.0205

**Table 6:** Repeatability error of extraction at different laser powers.

Method	Grayscale gravity	Proposed
Medium	1.8603	0.0178
Low	2.2814	0.0562

## Data Availability

Data are contained within the article. The data presented in this study are available at https://drive.google.com/drive/folders/1au9YMcu8aREACsHsyMniIA5kSUmhOurP?usp=drive_link (accessed on 15 October 2023).
